# RAD21 overexpression is frequently observed in BRCA-X Prostate Cancers

**DOI:** 10.1186/1897-4287-10-S2-A59

**Published:** 2012-04-12

**Authors:** S Deb, X Huiling, H Thorne, A Willems-Jones, D Clouston, D Bolton, R Ramsay, SB Fox

**Affiliations:** 1 Peter MacCallum Cancer Centre, Melbourne, Australia; 2 Victorian Cancer Biobank, Melbourne, Australia; 3 Kathleen Cunningham Foundation Consortium for Research into Familial Breast Cancer, Peter MacCallum Cancer Centre, Melbourne, Australia; 4 Focus Pathology, Melbourne, Australia; 5 Austin Health, Melbourne, Australia

## Background

RAD21 is a central component of the multi-protein cohesin complex which is important in several key cellular processes; apposition of sister chromatids, chromosomal segregation during mitosis and meiosis, error free homologous recombinational repair of DNA double strand breaks and epigenetic regulation of gene expression. Components of the cohesin complex are of the ‘BASC’, a BRCA1-associated multiprotein DNA repair complex. Aberrant RAD21 expression has been reported in several cancers with overexpression associated with more aggressive clinical course and chemo-resistance. In vitro cell lines and tumour xenografts of prostate cancers have shown overexpression of RAD21 compared with benign epithelium. Located at 8q24, this also appears to be a region often amplified in aggressive prostate cancer *in vivo.* We examined for RAD21 expression in familial prostate cancer.

## Methods

TMAs were created from 40 prostate cancer samples including 11 BRCA2 and 13 BRCAX associated tumours. IHC staining for RAD21 was correlated with E-cadherin, β-catenin, Androgen Receptor (AR), MUC1, AMACAR, Cyclin-D1 and clinico-histological factors.

## Results

53% of tumours overexpressed RAD21, with a higher proportion of BRCA-X tumours (80%, p=0.0082) compared with BRCA2 and sporadic cancers. RAD21 was positively correlated with Ki-67 (p=0.0053) and inversely with aberrant E-cadherin expression (p=0.0323). Within the sporadic/non-BRCA2/X tumour group, there was positive correlation with AR (0.0070) and Cyclin-D1 (p=0.0270).

## Conclusion

RAD21 expression is commonly present in prostate carcinoma and may be particularly important in the pathogenesis of BRCAX associated tumours. Within sporadic non-BRCA associated tumours, there is an association between RAD21 and AR expression, which may be biologically relevant and distinct in pathogenesis from those with aberrant E-cadherin expression.

